# Egocentric and allocentric spatial memory in healthy aging: performance on real-world tasks

**DOI:** 10.1590/1414-431X20198041

**Published:** 2019-04-15

**Authors:** C. Fernandez-Baizan, E. Diaz-Caceres, J.L. Arias, M. Mendez

**Affiliations:** 1 Department of Psychology, University of Oviedo, Oviedo, Spain; 2 Institute of Neurosciences of the Principality of Asturias, Oviedo, Spain; 3 Rehabilitation Services, Asturias Central University Hospital, Oviedo, Spain

**Keywords:** Egocentric spatial memory, Allocentric spatial memory, Aging, Spatial learning, Spatial strategies

## Abstract

Although normal aging has been related to several cognitive difficulties, other processes have been studied less, such as spatial memory. Our aim was to compare egocentric and allocentric memory in an elderly population using ecological tasks. Twenty-eight cognitively unimpaired participants performed Egocentric and Allocentric Spatial Memory Tasks, as well as Spatial Span from CANTAB, Benton's Judge of Line Orientation test (JoLO), and Montreal Cognitive Assessment test (MoCA). The results revealed that younger participants showed better performance than older participants on both the Egocentric and Allocentric Spatial Memory Tasks, although only the Egocentric test was able to discriminate between younger, middle, and older elderly participants. Learning effect was found in Allocentric Spatial Memory Task in younger and older groups, but not in the middle group. Allocentric and egocentric performance was not related to other visuospatial neuropsychological scores and gender did not influence performance in any task. Egocentric and Allocentric Spatial Memory Tasks may be useful tools in early screening for cognitive decline, as they are able to detect age differences in the cognitive unimpaired elderly population.

## Introduction

Aging is frequently associated with a reduction in cognitive abilities compared to adulthood, such as attention, working memory, free-recall long-term memory, and processing speed (1,2). These changes can be explained by age-related changes that normally occur in the brain regional volumes during aging, the integrity of white matter, and other structural and functional alterations (3).

Other important abilities for our daily lives, such as spatial memory and orientation, have been studied less in normal aging. Spatial orientation is the ability to find a trajectory to a target location through the environment without getting lost (4). It is a complex cognitive ability that requires the correct functioning of sensorial systems, visual perception, proprioception, memory, and the elaboration of plans (5). For spatial navigation, we use two types of strategies or frameworks. On the one hand, we employ the egocentric strategy, also known as path integration or dead reckoning (6), which specifies location and orientation with respect to the organism (7). Therefore, to orient ourselves using this framework, we need to be able to follow our own movements and to use our internal cues, as directions, distances, and turns from our own point of view (8). On the other hand, we use the allocentric strategy, also named place learning or cognitive mapping (9), which indicates location and orientation independent of the viewer's position, but with respect to environmental cues or elements, and eventually, all this information conforms visual and mental representations of our world (mapping) (9). For a completely functional spatial navigation, it is not enough to consider all these previous visuospatial or sensitive and proprioceptive cues, but it is necessary to switch, integrate, and combine them for creating global images of spatial representations (10).

Several conditions that affect mainly elderly people, such as mild cognitive impairment (11) or Parkinson's disease (12), have been related to worse performance in spatial memory tasks. Even spatial orientation performance could be a useful tool for detecting the earliest cognitive deficit of Alzheimer disease (AD) (13). One of the first brain areas affected in AD is the medial temporal lobe, including hippocampal and parahippocampal areas. The impairment of these areas has been related to a decline in cognitive functioning in elderly, especially in memory processes (14). All these areas have been linked with spatial orientation processing, mainly with allocentric strategy (15), but also with egocentric strategy (15). Therefore, it is not surprising that spatial memory is affected in AD.

This type of difficulty has also been found in normal aging, in comparison to younger adults. These results have been found especially in the allocentric framework and in switching from one strategy to another, so aging seems to be associated with egocentric-dependent navigation rather than allocentric (16). However, a great deal of research in spatial orientation was carried out using computer-based or virtual reality-based 2D tasks (17–20). In spite of being easier to administer, these tasks do not allow the participants to access somaesthetic, vestibular, and proprioceptive information (21,22). Therefore, these kinds of tests lack real world 3D information. The real world-based tasks are used less frequently and, when performed, they are not designed to compare the use of both orientation strategies in elderly (23) or they only compare different ages (24,25).

Gender has also been suggested as a relevant factor in spatial orientation achievement. Many studies have found that men usually outperform women (26), especially when the difficulty of the tasks is increasingly higher. However, other research found that the final performance depends on the type of cues, previous knowledge, or familiarity with the environment (27,28).

In the present study, the main goal was to compare the performance on egocentric and allocentric spatial orientation in an elderly non-cognitively-impaired population using real environment-based tasks. We expected differential effects of age and gender on results. We hypothesized that older groups in comparison to younger would have poorer ability in both orientation frameworks, whereas egocentric performance would outperform allocentric results in all age groups. We also expected an association between spatial orientation achievement and other visual and spatial abilities, such as forward and backward visuospatial span and judgment of line orientation.

## Material and Methods

### Participants

For the present study, 40 participants (16 males, 69.70±10 years old) were initially recruited (Table 1). Inclusion in the study required participants without neurological and psychiatric diseases or cognitive impairment. After administration of the Montreal Cognitive Assessment test (MoCA) (29), twelve of these participants were dismissed as they obtained scores lower than 26, the cut-off point proposed by the original authors. Finally, twenty-eight cognitively unimpaired participants (12 males, 71.25± 9.50 years old) completed the study. Based on percentiles of age (<33 and >66), subjects were divided into age groups: 62-66 years (5 females, 3 males), 67-74 years (5 females, 5 males), and 75 years or older (6 females, 4 males). All participants provided written informed consent, and the institutional review board approved the study (#178/16, Comité de Ética de la Investigación del Principado de Asturias).

**Table 1.** Descriptive characteristics of the sample.

Participants	N	MoCA	Total age	Age groups			Gender	
				62–66	67–74	75–81	Male	Female
Total	40	25.49±3.53	69.70±10	14 (35%)	15 (38%)	11 (27%)	16 (40%)	24 (60%)
Included	28	27.26±1.58	71.25±9.50	8 (28%)	10 (36%)	10 (36%)	12 (43%)	16 (57%)
Excluded	12	21.50±3.48	67.08±6.27	6 (50%)	5 (42%)	1 (8%)	4 (33%)	8 (67%)

Data are reported as means±SD or number and percent. MoCA: Montreal Cognitive Assessment test. Included participants are those that obtained 26 or higher scores in MoCA. Excluded participants are those that obtained 25 or lower scores in MoCA.

### Egocentric Spatial Memory Task

**Figure 1.**
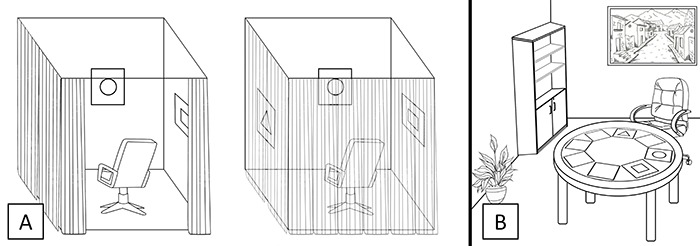
Representation of the experimental conditions of the Egocentric Spatial Memory Task (**A**) and the Allocentric Spatial Memory Task (**B**).

A task adapted from the Hashimoto test (30) was used for the assessment of the egocentric strategy (Ego). We examined the ability to represent spatial locations of objects placed on the walls around the subject. Each participant was placed inside 4 opaque panels to avoid environmental cues, forcing him/her to use body position as a reference. The task consisted of two parts. In part *A*, the participant stood in the center of a square surrounded by 4 panels and was instructed to remember the locations of three cards (circle, triangle, and cross), placed in one of the eight positions surrounding the subject (Figure 1). After 10 s, the examiner removed the cards and told the participant to put them back in their original location (10 seconds delay). In part *B*, the subject had to remember the locations of the same three cards. Immediately after the cards havd been removed, the subject was rotated to the right or to the left by 90 or 180°, as determined, and then asked to restore the three cards to the same position as before. For each part, a subject underwent 10 consecutive trials, earning 1 point for each card correctly located (full score on each part was 30 points).

### Allocentric Spatial Memory Task

A task designed to examine the ability to represent spatial locations of objects using distal spatial cues located in the room was used to assess the allocentric strategy (Allo). The participant was seated in a swivel chair around a round table with 8 possible locations, and he/she was instructed to remember the locations of the three cards (Figure 1). After 10 s, the participant was blindfolded, and the examiner moved the subject around the table to another position. From this new position, the participant was asked to restore the three cards to their original positions. Errors were corrected, showing the subject the correct position. The task consisted of 5 blocks of 4 trials. The position of the 3 cards on the table was the same in each block and repeated throughout its 4 trials, but the participant was moved to a different position in each trial. In each trial, the subject earned 1 point for each card correctly located (full score for each block is 12 points, 60 points in total).

### Neuropsychological tests

The cognitive screening was conducted with the Montreal Cognitive Assessment test (MoCA) (29), Spanish version. Visuospatial span and visuospatial working memory were assessed through the forward (Direct) and backward (Inverse) variant, respectively, of Spatial Span (SSP-D and SSP-I) from the Cambridge Neuropsychological Assessment Battery (CANTAB) (31). This neuropsychological battery was selected because it had been previously employed for cognitive assessment in healthy aging (32). Visuospatial ability was evaluated with Benton's Judge of Line Orientation test (JoLO) TF 2/3 H11-30 (33).cs

### Statistical analysis

Descriptive analyses were used to show means and standard deviations of every dependent variable. In this exploratory study, analyses of variance (ANOVA) were done to compare age groups and/or gender differences. For *post hoc* analysis, Holm-Sidak tests were applied. Paired sample *t*-tests were used to compare egocentric and allocentric scores. Repeated measures ANOVAs were used to assess performance improvement across trials in Allocentric Task. Finally, bivariate correlation analysis was done to assess relationships between spatial orientation tasks and neuropsychological variables. Analyses were carried out with the SigmaStat software version 3.2 (Systat, USA). Results with P<0.05 were considered statistically significant.

## Results

The mean scores and standard deviations for all the tests are reported in Table 2.

**Table 2.** Mean and standard deviation for test scores across age groups.

Tasks	62-66 years (n=8) Mean (SD)	67-74 years (n=10) Mean (SD)	>75 years (n=10) Mean (SD)
MoCA	28.13 (1.64)^a^	27.40 (1.90)	26.20 (0.42)
EGOA	29.50 (1.07)^a^	26.80 (4.02)	26.20 (1.81)
EGOB	29.50 (6.23)^b^	21.20 (3.97)^c^	17.00 (7.07)
ALLO	51.00 (5.53)^b^	42.30 (6.77)	34.70 (13.30)
JoLO	24.75 (4.16)	27.00 (3.68)	27.40 (2.63)
SSP-D	5.50 (1.07)	5.20 (0.63)	4.90 (0.79)
SPP-I	4.63 (0.74)	4.70 (0.82)	4.80 (0.91)

MoCA: Montreal Cognitive Assessment test; EGO A: Egocentric Spatial Memory Task part A; EGO B: Egocentric Spatial Memory Task part B; ALLO: Allocentric Spatial Memory Task; JoLO: Benton's Judge of Line Orientation test; SSP-D: Spatial Span Direct; SSP-I: Spatial Span Inverse (^a^P<0.05, 62-66 years compared with >75 years; ^b^P<0.001, 62-66 years compared with >75 years; ^c^P<0.05, 67-74 years compared with 62-66 years; ANOVA).

Our data showed significant differences between the groups in Ego A (F(2,25)=33.632; P=0.041), Ego B (F(2,25)=10.223; P<0.001), Allo (F(2,25)=6.659; p=0.005), and MoCA (F(2,25)=4.074; P=0.029). In Ego A Task, we found differences between the youngest (62-66 years) and oldest (75-81 years) groups, the youngest showing better scores compared to the oldest (t=2.570; P=0.049). Similarly, youngest and oldest participants differed in Ego B Task score (t=4.484; P<0.001), but this task also revealed significant differences between participants who were 62-66 years old and 67-74 years old (t=2.977; P=0.013). In the Allo Task, the youngest participants outperformed the oldest (t=3.648; P=0.004). Similar results were also found in MoCA (t=2.791; P=0.029).

We did not find differences between age groups on JoLO (P=0.257), SSP-D (P=0.313), and SSP-I (P=0.906), using ANOVA.

Gender did not reveal significant differences in any task: Ego A (P=0.174), Ego B (P=0.541), Allo (P=0.174), MoCA (P=0.146), SSP-D (P=0.074), SSP-I (P=0.631), and JoLO (P=0.397) with ANOVA. Similarly, our data failed to show significant differences for age × gender in Ego A (P=0.820), Ego B (P=0.697), Allo (P=0.672), MoCA (P=0.626), SSP-D (P=0.612), SSP-I (P=0.953), or JoLO (P=0.107), using two-way ANOVA.

**Figure 2.**
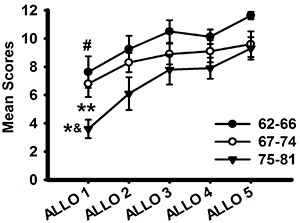
Mean scores of the groups in the allocentric trials (1 to 5). Significant differences were found in the first trial between the 62-66 and 75-81 groups of age (*P=0.014), as well as between the 67-74 and 75-81 groups of age (^&^P=0.03). Improvement across trials was also found in the 62-66 group, with a lower score in the first trial compared to the fifth trial (^#^P=0.003). The 75-81 group also showed an improvement across learning trials, with a lower score in the first trial than in the rest (second to fifth) (**P<0.001). Data are reported as means±SD (ANOVA).

Performance of each group across Allocentric Spatial Memory trials is shown in Figure 2. Our data revealed a significant effect of learning in successive trials of Allo Task in the youngest group (62-66) (F(4,28)=34.727; P<0.001), but this difference was found only between first and fifth trial (t=10.872; P=0.003) (repeated measures ANOVA). We also found significant differences between trials of the Allo Task in the oldest group (75-81) (F(4,36)=34.727; P<0.001) using repeated measures ANOVA. First trial score was lower than second, third, fourth, and fifth (P<0.001) in *post hoc* test. The middle group (67-74) showed no improvement across learning trials, as trial comparison did not reveal significant differences (P=0.114). Comparing trials between age groups, our data showed significant differences only in the first trial of Allo Task (F(2,25)=34.727; P=0.010) between 62-66 group and 75-81 group (t=3.095; P=0.014), and between 67-74 group and 75-81 group (t=3.200; P=0.030). No differences were found between 62-66 and 67-74 years of age (P=0.532).

Comparing scores of the whole sample in Ego and Allo Tasks with paired *t*-test analysis, our data showed significant differences between these two strategies (t(27)=-3.418; P=0.002) with a higher performance on egocentric strategy. However, no significant differences were found when allocentric and egocentric strategies were compared in younger (P=0.069), middle (P=0.182), or older groups (P=0.105).

A significant interaction was observed between MoCA and Allo (R=0.399; P=0.035, Pearson's correlation analysis).

## Discussion

The aim of this study was to introduce for the first time novel tasks that recreate the natural conditions of spatial memory as far as possible. To our knowledge, this is the first study in non-pathological aging that assesses egocentric and allocentric frameworks separately in order to extend the knowledge about spatial memory in this population employing real-world-based tasks.

Our data demonstrated that the elderly population without cognitive impairment or dementia showed different performance on spatial orientation, as well as on general cognitive capacity, at different ages. These results are in line with previous studies, which reported that spatial orientation is progressively impaired with aging (34). Spatial orientation achievements seem to show an “inverted U” curve during development, reaching the highest scores at adolescence and adulthood, while worse performance is found during childhood and old age (25). These results could be due to functional changes in the elderly brain. In aging, there is a reduction in the activation of the hippocampus and parahippocampal gyrus, as well as the retrosplenial cortex and parietal lobe (35). All of these brain regions are involved in spatial orientation performance.

Although results pointed out that egocentric and allocentric performance was different in the whole sample, we found that the performance on the egocentric and allocentric strategies was quite similar in each age group. Surprisingly, it seems that in different stages of old age there was no preference for allocentric framework over egocentric. These results contrasted with previous studies showing that, compared to adults, the elderly tended to use the egocentric framework instead of the allocentric (16). Although the classical neural bases of the egocentric and allocentric frameworks are different (16), there are more areas that participate in spatial navigation, which seem to overlap in both strategies: frontal, parietal, temporal, occipital cortex, and cerebellum (15). This points out that the lack of preference for one strategy over the other one could be due to the regular brain degeneration associated with aging, which could affect the same areas that control both spatial frameworks.

We also found that spatial memory seemed to be more affected at older ages, although strategies appeared to be altered at different moments in the elderly: allocentric strategy performance was intact from 62 to 74 years of age, but the egocentric strategy declined from 62 to 66 years old and continued to decline from 67 to 75 years old. This suggested that the egocentric orientation framework was affected earlier in old age, whereas the allocentric orientation framework was preserved longer. These results could be explained by methodological variables or by brain connectivity. First, we know that egocentric encoding is not just remembering the position of the objects, taking one's body as a reference, but it also involves updating the distance, representing the speed, and updating our self-movement (8). Previous studies usually employed virtual tasks or 2D tasks, without any participant movement during them (18 [Bibr B19],^20^,^36^). However, when functional and ecological tasks are employed, the results point out that egocentric strategy begins to decrease from 60 years of age (25) or even does not show a difference at 80 years of age compared to young adults (24), whereas decline on allocentric strategy occurs at around age 70 (24,25). Therefore, our task was more similar to the real conditions of spatial navigation because it included movement of the body and could detect problems in egocentric strategy performance that virtual tasks could not. Besides, neurofunctional image studies show that the fronto-parietal network, which is involved mainly in egocentric strategy**,** is progressively impaired in the elderly (37). In addition, it has been found that posterior parietal cortex, which participates in both fronto-parietal network and egocentric framework, processes visual, vestibular, auditory, and somatosensory information (15), that as we mentioned before, are indispensable for egocentric navigation. To sum up, the earlier decline of egocentric strategy instead of allocentric could be related with premature functional changes associated with aging.

The results showed a significant learning effect during Allocentric Spatial Memory Task in the younger and older elderly group, but not in the middle group. Although the middle group did not show differences with respect to the younger group in total scores in the Allocentric Spatial Memory Task, we can observe by assessing the progression across learning trials that they did not learn as fast as the younger group did. Therefore, allocentric learning seemed to be impaired from 67 years of age, while allocentric performance was not affected until age 75. This could be due to earlier brain function decline that seems to affect in a softer way than egocentric strategy. Improvements in allocentric strategy between 62 and 67 years of age were only found between the first and the last trial. Participants in this group started scoring around the mean and were progressively increasing their efficiency up to the last trial, where they nearly reached ceiling effect. However, between 75 and 81 years of age, improvements were found in almost every trial. They began the tasks with quite poor scores but were getting better results in every new trial. At these older ages, the results achieved in the last trial were easily reached by the youngest in the second trial. The most positive conclusion of this study is that spatial learning ability is not completely impaired in the most elderly sample. This conclusion is in line with previous data (24). Therefore, spatial learning does not seem to be entirely impaired by aging processes in cognitively normal adults, contrary to clinical populations as AD.

Contrary to what was expected, we did not find gender differences in any task. Nevertheless, almost all the studies about spatial performance were done exclusively in men and they were performed in young and adult subjects (34,38). Therefore, their results are not directly comparable with the results presented here. When men and women performances were compared in elderly participants, contradictory results were found. León et al. (18) and Tascón et al. (26) found that men in the late adulthood outperform woman, while Gazova et al. (24) detected non-differences between genders. Other studies, which assessed participants' hormonal levels, suggested the role of testosterone in spatial memory improvement in males, even though levels of this sex hormone decrease with aging (17). Therefore, the absence of gender differences in our results could be due to the effect of hormonal variables non-controlled in the present study.

We found that other spatial measurements, such as spatial span, spatial working memory, and recognition of line orientation, usually employed in neuropsychology assessments, do not discriminate between different ages in elderly people without mild cognitive impairment or dementia. However, on part A of the Egocentric Spatial Memory Task, which is based on a spatial span task with only three items to remember, we found differences between the youngest and the oldest group of participants. Therefore, three-dimensional memory tasks seem to be more useful than classical tasks for the assessment of the elderly.

Correlation results showed that Allocentric and Egocentric Spatial Memory Tasks were not linked with other visuospatial measures, contrary to our hypothesis, although MoCA and Allocentric Spatial Memory Task scores seemed to be related. Our results partially agree with previous studies (25,39), which found that neither visuoperceptive functions nor general cognitive functioning affect spatial orientation. However, our findings could suggest the Allocentric Spatial Memory Task as a useful tool for complementary assessment of spatial deficit associated with cognitive decline, or in those cases where spatial skills need to be assessed in depth. Previous studies on allocentric strategy found that decline of orientation ability in elderly people is exclusively found in new-route learning but not in well-known environments (23), assuming the role of the hippocampus and medial temporal lobe in the formation of new spatial memories. Therefore, if we manipulate familiarity of the allocation where Allocentric Spatial Memory Task is conducted, we can easily detect medial temporal lobe damage as well, as it occurs in mild cognitive impairment or AD.

The present study had limitations. The size of the sample and the absence of some important variables in aging, such as medicine intake, could have influenced the results. Despite this, the Egocentric and Allocentric Spatial Memory Tasks used in this study could become a promising tool for the assessment of spatial memory performance, especially in those cases where visuospatial skills need to be assessed in depth. Further studies are required to explore the performance on these tasks in other populations.
